# Prevalence of helicobacter pylori infection among children living in a rural setting in Sub-Saharan Africa

**DOI:** 10.1186/s12889-017-4274-z

**Published:** 2017-04-24

**Authors:** Yaw Asante Awuku, David Larbi Simpong, Ishmael Kunateh Alhassan, Derek Anamaale Tuoyire, Taiba Afaa, Patrick Adu

**Affiliations:** 1 Department of Medicine and Therapeutics, University of Cape Coast, Cape Coast, Ghana; 2 Department of Medical Laboratory Technology, University of Cape Coast, Cape Coast, Ghana; 3 Department of Community Medicine, Universty of Cape Coast, Cape Coast, Ghana; 4 Department of Child Health, University of Ghana, Accra, Ghana

**Keywords:** H.pylori infection, Prevalence, Immune chromatographic assay, Children and Ghana

## Abstract

**Background:**

Helicobacter pylori infection affects more than half of the world’s population. It is generally acquired during childhood with no symptoms but has long- term clinical sequelae. This study estimated the prevalence of H. pylori infection amongst children in a rural environment in Africa.

**Methods:**

We conducted a cross-sectional study over a four (4)-month period within two rural communities. 240 asymptomatic children were tested using lateral flow immunochromatographic assay for the qualitative detection of H. pylori antigen in a fecal specimen. Statistical analysis and processing was done using Stata version 11.

**Results:**

The mean age of the participants was 10.5 ± 2.7 years with the predominant age range being 8–10 years (34.6%), and a mean household size of 7.1 ± 1.7. The study population showed a female preponderance of 57.1%. 88% of the H. pylori positive children lacked pipe and borehole drinking water. All of the positive H. pylori children practiced open-air defecation. The overall prevalence of H. pylori infection among children in this study was at least 14.2%.

**Conclusion:**

Our study demonstrated a high prevalence of H. pylori infection among children in a rural setting. Educational status of parents did not affect H. pylori prevalence but increasing household numbers, female gender, source of drinking water other than pipe and borehole, open-air defecation and younger age were associated with a higher H. pylori prevalence.

## Background

*Helicobacter pylori (H. pylori)* is an important global infection with a worldwide prevalence of about 50 % [1, 2]. This infection is mostly acquired during childhood through the fecal-oral and oral-oral route [3, 4]. Initial infection with this organism is usually silent but symptoms and pathologic changes occur later in life. The clinical conditions and pathologic changes associated with *H. pylori* infection include gastritis, gastric and duodenal ulcers, gastric cancers, iron deficiency anaemia and idiopathic thrombocytopenic purpura (ITP) [5–8]. *H. pylori* infection exhibits a varied geographic distribution on both local and global scales. These variations are mostly socioeconomically driven; factors such as age, gender, genetic predisposition, ethnicity, educational level and sanitation determine the incidence and prevalence of this global infection [9–11].

The diagnosis of H. pylori infection is generally based on methods described as invasive or non-invasive approaches. The invasive methods are based on endoscopy and biopsy using modalities like culture, histology and rapid urease test. Non-invasive diagnostic methods include urea breath test (UBT), fecal antigen testing, serology and more recently polymerase chain reaction testing of feces. The sensitivities of the invasive and non-invasive tests are comparable and relevant in clinical practice [12, 13]. This study used the lateral flow monoclonal immune-chromatographic testing (ICT) technique to determine the prevalence of *H. pylori* among children.

The primary objective of this study was to determine the prevalence of H. pylori infection among the pediatric population in a rural West African setting.

Additionally, we evaluated the sociocultural and demographic factors influencing the prevalence of this infection in the communities.

## Methods

### Study population and design

This cross-sectional study was conducted from December 2014 to March 2015 within two rural communities in the Upper West region of Ghana. The Upper West is located at the North western corner of Ghana with latitude 9.8° - 11.0° north and longitude 1.6° - 3.0° west, bounded to Burkina Faso to the north. A total of 240 children (≤16 years) were recruited for the study. This was done by assigning the students’ numbers 1 and 2 with all those numbered 2 selected for this study. Informed consent was obtained through community leaders, teachers and parents of those who participated in the study.

### Demographic details

The age, gender, type of toilet facility, source of drinking water, number of people in their household, number of siblings, and the educational level of the children as well as their parents were collected from all participants using a structured questionnaire.

### *Helicobacter pylori* testing

Participants were given clean stool sample containers to provide fresh stool samples within 2 h in the sterilized containers provided.The children were asked to indicate the time and date of specimen collection. As an exclusion criteria, any specimen that lasted for more than 2 h before reaching us was discarded. All stool specimen were collected during their morning (7:00 am) assembly section and transported in a cold (−4 °C) specimen carrier to the laboratory. Specimen that we anticipated will stay more than 2 h before processing (from time indicated on the container and the time of testing) in the laboratory were refrigerated at (−20 °C). Eight samples in all were discarded with reference to the exclusion criteria defined in sample collection.

A lateral flow immunochromatographic assay for the qualitative detection of *Helicobacter pylori* antigen in human fecal specimen (*OnSite H. pylori* Ag Rapid Test, USA) was used.

### Statistics

The data was captured using Microsoft excel, 2007 and exported to STATA version 11.0 for processing and analysis. Univariate and bivariate analysis were used to describe the results in the form of frequency and percentage distributions. Pearson’s chi squared and Fisher’s exact tests employed to test associations between various background characteristics of participants and the outcome of interest (*H. pylori* status) at *p* < 0.05 significance level.

## Results

### Characteristics of study population

The participant characteristics are as presented in Table 1. The mean age of the participants was 10.5 ± 2.7 years. The predominant age range was 8–10 years constituting 34.6%. However, the age group with the highest incidence of *H. pylori* infection was 5–7 years that had 19.5% positivity. The age group with the least *H. pylori* infection rate was 14–16 years with prevalence of 11.9%. The study population showed a female: male ratio of 1.3:1, with a higher proportion of females having *H. pylori* infection compared to males [16.8% (females) vs 10.7% (males)].

**Table 1:** Distribution of sample characteristics

	H. Pylori Status			
	Negative	Positive		
Characteristic	N (%)	N (%)	Total	95% CI
Participants	206 (85.8)	34 (14.2)	240	0.097, 0.186
AGE				
5–7	33 (80.5)	8 (19.5)	41	0.068, 0.321
8–10	73 (87.9)	10 (12.1)	83	0.048, 0.191
11–13	63 (85.1)	11 (14.9)	74	0.656, 0.231
14–16	37 (88.1)	5 (11.9)	42	0.016, 0.221
Pearson chi2(1) = 1.4762 Pr = 0.688				
SEX				
Female	114 (83.2)	23 (16.8)	137	0.104, 0.231
Male	92 (89.3)	11 (10.7)	103	0.461, 0167
Pearson chi2(1) = 1.8044 Pr = 0.179				

Table 2 stratifies the study participants with regards to *H. pylori* infection and its association with educational level of parents and household size. The educational level of either parent was not significantly associated with *H. pylori* infection rate. The mean household size of the study participants was 7.1 ± 1.7. Interestingly, participant household size was significantly associated with the prevalence of *H. pylori* infection (*p* = 0.000; Fisher’s exact test).

**Table 2:** Association of H. pylori infection with parent education and household size

	H. Pylori Status			
	Negative	Positive		
Characteristic	N (%)	N (%)	Total	95% CI
Mother’s Edu. Status				
No	170 (85.9)	28 (14.1)	198	0.092, 0.190
Yes	36 (85.7)	6 (14.3)	42	0.032, 0.253
Pearson chi2(1) = 0.0006 Pr = 0.981				
Father’s Edu. Status				
No	140 (86.4)	22 (13.6)	162	0.824, 0.189
Yes	66 (84.6)	12 (15.4)	78	0.071, 0.235
Pearson chi2(1) = 0.1410 Pr = 0.707				
No. of People in a Household				
4	11 (84.6)	2 (15.4)	13	0.073, 0.380
5	20 (60.6)	13 (39.4)	33	0.217, 0.569
6	22 (56.4)	17 (43.6)	39	0.273, 0.598
7	51 (96.2)	2 (3.8)	53	0.015, 0.090
8	43 (100)	0	43	-
9	41 (100)	0	41	-
10	18 (100)	0	18	-
Fisher’s exact test: Pr = 0.000				

Table 3 stratifies the *H. pylori* infection status of the participants with regards to number of siblings and lifestyle choices. Overall, 14.2% of the participants had *H. pylori* infection. About 48.3% of the participants had no access to pipe/borehole as a source of water. Whereas 25.9% (116/240) of those having no access to pipe/borehole were *H. pylori* positive, only 3.2% of those with pipe/borehole were *H. pylori* positive. This difference was statistically significant (*p* = 0.000; Fisher’s exact test). Overall, 88% (30/34) of the H. pylori stool antigen positive participants lacked pipe/borehole drinking water. In addition, 70.4% (169/240) of the participants practiced open air defaecation. Not surprisingly, 20.1% of those practicing open-air defaecation were *H pylori* positive, none of the participants using pit/wc was *H. pylori* positive.

**Table 3:** Association of *H. pylori* infection with lifestyle choices

	H. Pylori Status			
	Negative	Positive		
Characteristic	N (%)	N (%)	Total	95% CI
Number of siblings				
1–3	103 (75.2)	34 (24.8)	137	0.174, 0.321
4+	103 (100%)	0	103	−
Fischer’s exact test: *p* = 0.000				
Pets/Livestock				
None	62(64.6)	34 (35.4)	96	0.256, 0.451
Pet	49 (100)	0	49	−
2 livestock	95 (100)	0	95	−
Fischer’s exact test: *p* = 0.000				
Source of drinking water				
Pipe/borehole	120 (96.8)	4 (3.2)	124	0.001, 0.063
Other than pipe/borehole	86 (74.1)	30 (25.9)	116	0.177, 0.339
Fisher’s exact test: Pr = 0.000				
Type of laterine facility				
Open air	135 (79.9)	34 (20.1)	169	0.140, 2.262
Pit/WC	71 (100)	0	71	−

## Discussion

*H. pylori* infection affects more than half of the world’s population including children. Because of the fecal-oral mode of transmission it is more prevalent in poor socioeconomic environments. H. pylori is described in current medical literature as a group 1 carcinogen but the health implications of infection in children remains uncertain [2]. This may be problematic particularly in African children who may harbor more virulent strains. The long term sequelae of H. pylori infection was ignored because of what has been described as “African enigma” [6]. It has however been shown that all complications including carcinoma do occur in the African population [6, 14]. Our study used a noninvasive method to qualitatively detect *H. pylori* antigen in human fecal specimen. We acknowledge the limitation in testing as the *H. pylori* stool antigen testing was not compared with any gold standard diagnostic method. However, the ethical and feasibility challenges of upper gastrointestinal endoscopy as a screening tool in an asymptomatic pediatric population was strongly considered [15].

The global prevalence of H. pylori infection is generally higher in developing countries than the developed world [16, 17]. Transmission of *H. pylori* infection is rapidly decreasing in the developed countries predictably because of improvement in the sanitation [18, 19]. There is infrequent *H. pylori* infection during childhood in the developed countries with the United States reporting less than 5% in under five years and up to 10% by adolescent age. However in developing countries the story is different with about 50% of children under five infected and as high as 90% in some adult populations [20, 21]. We demonstrated a prevalence of at least 14.2% among asymptomatic children between the ages of 5–16 years living in a rural setting in sub-Saharan Africa (SSA). Our finding in terms of prevalence of H. pylori infection among children was not consistent with the developing countries data [22–24]. This could be attributed to the differences in the study design, population dynamics, as well as the specificities of techniques employed in the studies. Antibody testing procedures for *H. pylori* detection are used in some studies but is unable to differentiate active infection from a previous in the early weeks of follow up after eradication therapy. Our study employed *H. pylori* stool antigen testing methods because of its non-invasive nature especially in a children population.

Our study had a female preponderance of 57.1% with higher H. pylori prevalence among females (16.8%) than males (10.7%). The gender effect on prevalence of H. pylori infection in many populations is varied. Woodward and colleagues reported a higher prevalence in men than women [20, 23] whiles others reported no difference. A meta-analysis reported a male predominance in adults but not in children [19, 25, 26]. Our study in children demonstrated a higher prevalence in females with no plausible explanation.

The age effect on prevalence is well documented in H. pylori epidemiology with a positive correlation between age and prevalence [27, 28]. Our study showed higher prevalence in the younger age group and decreasing prevalence with advancing age. This is a reverse of the pattern in most population studies. Educational status has been used as a proxy marker of socioeconomic status and an important determinant of *H. pylori* prevalence in developed and resource limited settings [29–31]. The educational status of parents in our study did not affect the prevalence significantly. Crowding in households and increasing household contact have been linked as risk factors of *H. pylori* infection [32, 33]. As the number of people in a household increased the prevalence of *H. pylori* also increased [34]. Source of drinking water other than pipe and borehole increased the prevalence of *H. pylori* infection as well as open-air defecation. Water is an important source of H. pylori spread and many studies have actually confirmed it. Therefore, handling of water and poor sanitation will be a good milieu for the spread of this infection. Our study support this assertion as we also realized that source of water other than pipe and borehole and open air defecation were associated with higher prevalence of H. pylori infection [35, 36].

In our environment, poverty and poor socioeconomic status are associated with higher household numbers. It is therefore not surprising giving the mode of transmission that prevalence in our study increased with increasing household numbers. Proper hand washing and waste disposing systems will help control infection in households especially in crowded places.

## Conclusion

Our study demonstrated a high prevalence of H. pylori infection among children in a rural setting. Educational status of parents did not affect H. pylori prevalence but increasing household numbers, female gender, younger age, open-air defecation and source of drinking water other than pipe and borehole were associated with a higher prevalence.

## Abbreviations

ICT: Immune chromatographic testing
ITP: Idiopathic thrombocytopenic purpura
UBT: Urease Breath Test
